# The Landscape of Expressed Chimeric Transcripts in the Blood of Severe COVID-19 Infected Patients

**DOI:** 10.3390/v15020433

**Published:** 2023-02-04

**Authors:** Sunanda Biswas Mukherjee, Rajesh Detroja, Sumit Mukherjee, Milana Frenkel-Morgenstern

**Affiliations:** 1 Cancer Genomics and BioComputing of Complex Diseases Lab, Azrieli Faculty of Medicine, Bar-Ilan University, Safed 1311502, Israel; 2 Princess Margaret Cancer Center, University Health Network, Toronto, ON M5G 2C4, Canada; 3 National Cancer Institute (NCI), National Institutes of Health (NIH), Bethesda, MD 20892, USA

**Keywords:** chimeric transcripts, severe COVID-19, aberrant splicing, cis-SAGe, respiratory viral infections

## Abstract

The ongoing COVID-19 pandemic caused by SARS-CoV-2 infections has quickly developed into a global public health threat. COVID-19 patients show distinct clinical features, and in some cases, during the severe stage of the condition, the disease severity leads to an acute respiratory disorder. In spite of several pieces of research in this area, the molecular mechanisms behind the development of disease severity are still not clearly understood. Recent studies demonstrated that SARS-CoV-2 alters the host cell splicing and transcriptional response to overcome the host immune response that provides the virus with favorable conditions to replicate efficiently within the host cells. In several disease conditions, aberrant splicing could lead to the development of novel chimeric transcripts that could promote the functional alternations of the cell. As severe SARS-CoV-2 infection was reported to cause abnormal splicing in the infected cells, we could expect the generation and expression of novel chimeric transcripts. However, no study so far has attempted to check whether novel chimeric transcripts are expressed in severe SARS-CoV-2 infections. In this study, we analyzed several publicly available blood transcriptome datasets of severe COVID-19, mild COVID-19, other severe respiratory viral infected patients, and healthy individuals. We identified 424 severe COVID-19 -specific chimeric transcripts, 42 of which were recurrent. Further, we detected 189 chimeric transcripts common to severe COVID-19 and multiple severe respiratory viral infections. Pathway and gene enrichment analysis of the parental genes of these two subsets of chimeric transcripts reveals that these are potentially involved in immune-related processes, interferon signaling, and inflammatory responses, which signify their potential association with immune dysfunction leading to the development of disease severity. Our study provides the first detailed expression landscape of chimeric transcripts in severe COVID-19 and other severe respiratory viral infections.

## 1. Introduction

The ongoing COVID-19 pandemic is caused by SARS-CoV-2, a recently identified member of the Coronaviridae family [1,2]. The clinical representation of COVID-19 is variable, and sometimes it can lead to the development of disease severity resulting in immune dysfunction, acute respiratory distress syndrome (ARDS), and multi-organ failure [3,4,5]. Despite several studies, the molecular mechanisms behind the development of severe COVID-19 are still unclear. During the severe COVID-19 infection, SARS-CoV-2 antagonizes interferon (IFN) induction and IFN signaling, which generates dysfunctional immune response [6,7,8,9,10,11,12]. Recent studies demonstrated that SARS-CoV-2 suppresses the interferon (IFN) response by disrupting the host cell splicing [13]. Aberrant splicing and dysregulation of transcripts were found to be strongly correlated with the clinical severity of COVID-19 [14,15].

Aberrant splicing promotes the increase in non-canonical splicing events, resulting in the generation of novel chimeric transcripts [16,17,18,19]. In several cancers and complex diseases, non-canonical splicing was observed, which was reported to generate novel chimeric transcripts [18,20,21]. Chimeric transcripts are formed by the fusion of the exon/intron from two separate genes [22]. The generation of chimeric transcripts was found to be functionally involved with the development of several diseases [23,24]. Further, the appearance of chimeric transcripts in the cell is thought to be associated with the generation of phenotypic plasticity that helps the cell’s survival in response to specific stress [25,26]. Chimeric transcripts could modify cell functionality by altering the regulatory network and protein interaction network, and they can also switch pathways [27,28,29].

SARS-CoV-2 induces the widespread alternation of host cell-splicing machinery and transcriptional dynamics in severe COVID-19 infection, which raises the possibility of the generation and expression of novel chimeric transcripts in severe COVID-19-infected cells. However, no study so far has attempted to check if SARS-CoV-2-induced aberrant splicing in severe COVID-19 infections could generate novel chimeric transcripts, nor the potential functional impact of these chimeric transcripts’ generation. In this study, we integrated publicly available RNA-seq datasets of blood from 85 severe COVID-19 patients, 10 mild COVID-19 patients, 84 other severe respiratory viral infected patients, 54 healthy persons, and 199 samples from 32 different tissues and identified the chimeric transcripts unique to severe COVID-19 infections.

## 2. Materials and Methods

### 2.1. Acquisition of the RNA-Seq Datasets

RNA-seq data from several sources were collected for this study. A total of 44 blood samples of RNA-seq data from severe COVID-19 patients and 10 samples from healthy donors were obtained from the Gene Expression Omnibus (GEO) database (GSE171110) [30]. RNA-seq data for 24 severe COVID-19 and 34 healthy blood samples were obtained from the GSE152418 dataset [31]. We also collected single-cell RNA sequencing data of peripheral blood mononuclear cells (PBMCs) from 8 severe COVID-19 patient samples and 6 healthy controls (PRJNA633393) [32]. We downloaded the RNA-seq data of blood from EBI ArrayExpress (accession E-MTAB-10926) for 10 mild COVID-19 patients and 10 severe COVID-19 patients [33]. From the GSE157240 dataset, we downloaded the RNA-seq data for 84 blood samples of patients with severe respiratory viral infections caused by influenza, enterovirus/rhinovirus, human metapneumovirus, dengue virus, cytomegalovirus, Epstein–Barr virus, or adenovirus was also obtained [34]. Further, we downloaded RNA-seq data from 199 samples from 122 individuals representing 32 different healthy human tissues from EBI ArrayExpress (accession E-MTAB-2836). The downloaded raw sequence reads were converted to FASTQ using the SRA toolkit, version 2.10.7 [35].

### 2.2. Identification of Chimeric Transcripts from the RNA-Seq Data

Using our in-house reference-based method ChiTaH [36], all the above samples were used to identify chimeric transcripts. Recently, ChiTaH [36] was demonstrated to be the most efficient reference-based approach for chimeric transcript detections from RNA-seq data compared to other popular tools such as EricScript [37], STAR-Fusion [38], JAFFA [39], and FusionCatcher [40]. Furthermore, ChiTaH can also detect the chimeric transcripts from single-cell RNA (scRNA)-seq data. ChiTaH maps RNA-Seq datasets and predicts potential chimeric transcripts in each sample using 43,466 non-redundant high-quality human chimeras from the ChiTaRS 5.0 database [41]. The three rigorous criteria listed below were used to determine which transcripts were chimeric: (i) Reads should only map with human chimeric transcripts found in the ChiTaRS database and not with the human genome or transcriptome, (ii) at least five reads should cover the chimeric transcript junction length, and (iii) the mapping reads’ quality should be MAPQ > = 10.

### 2.3. Differential Gene Expression Analysis

STAR aligner [42] was used to map the RNA sequencing reads from all samples; the human reference genome (hg38) was used for alignment. Next, the featureCounts tool [43] was used to determine the total amount of reads mapped to each human gene. Finally, DEseq2 [44] was used to perform differential gene expression analysis. When log2foldchange ≥ 2 and *Padj* ≤ 0.05, a gene was deemed significantly upregulated, and when log2foldchange was negative and *Padj* ≤ 0.05, it was deemed significantly downregulated. The Benjamini–Hochberg method was used to adjust the *p*-values and the false discovery rate (FDR) [45].

### 2.4. Gene Ontology and Pathway Enrichment Analysis of the Parental Genes of Chimeric Transcripts

Metascape [46] was used to perform the Gene ontology (GO) biological process and pathway enrichment analyses. The *p*-value cutoff < 0.05 was set to select the enriched processes and pathways. The KEGG [47] and REACTOME [48] pathway databases were used during the pathway enrichment analysis.

## 3. Results

### 3.1. Identification of Chimeric Transcripts in the RNA-Seq Data of Blood Samples from Severe COVID-19, Mild COVID-19, and Other Severe Respiratory Virus-Infected Patients

**Figure 1:**
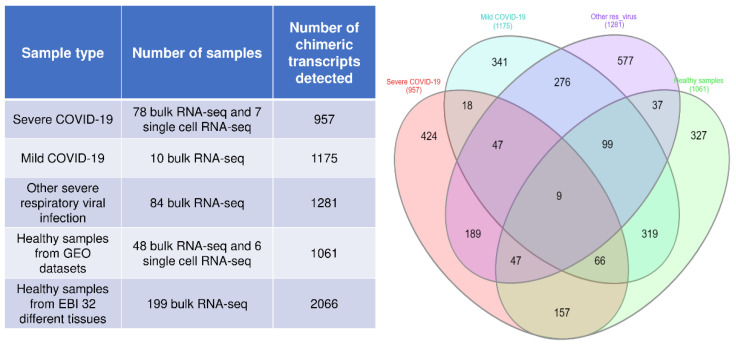
The table represents the number of chimeric transcripts detected in the different samples. The Venn diagram represents the common and unique chimeric transcripts detected in each sample.

We identified a total of 957 chimeric transcripts from 78 bulk RNA-seq and 7 single-cell RNA (scRNA)-seq data from severe COVID-19 patients. In total, 1175 chimeric transcripts were identified in mild COVID-19 patients from 10 bulk RNA-seq samples, and 1281 chimeric transcripts were identified in various severe respiratory viral infections from 84 bulk RNA-seq samples. Moreover, we also identified 1061 chimeric transcripts from 54 healthy samples, while 2066 chimeric transcripts were identified in 199 samples from 32 normal tissues. Chimeric transcripts were observed to be abundant in all samples analyzed (Table S1 (Supplementary Materials)). However, from a pathological perspective, it would be interesting to identify the chimeric transcripts that are specific to severe COVID-19 infections, as well as those that are commonly expressed across multiple severe viral infections. We have identified that 44.3% (424 out of 957) of chimeric transcripts identified in the severe COVID-19 samples are unique to severe COVID-19 infections (Figure 1). Furthermore, we have identified 189 chimeric transcripts, which are common to severe COVID-19 infection and other severe respiratory viral infections. These results indicate that a significant number of chimeric transcripts detected in individuals are common to severe COVID-19 and other severe respiratory viruses.

### 3.2. Identification of Severe COVID-19 Specific Recurrent Chimeric Transcripts and Functional Analysis of Their Parental Genes

**Figure 2:**
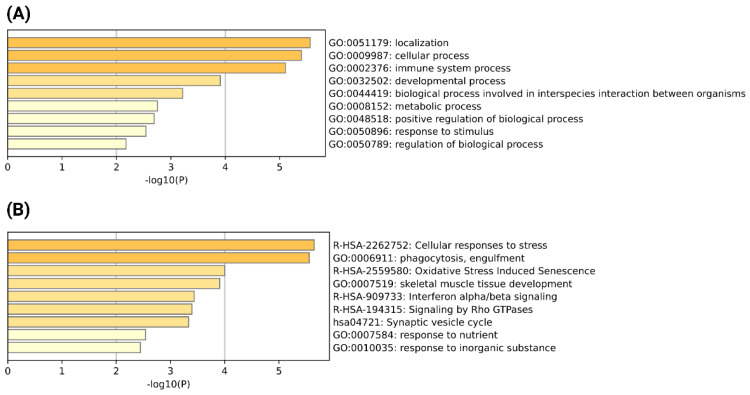
(**A**) GO biological process enrichment analysis of the parental genes of severe COVID-19 specific recurrent chimeric transcripts; (**B**) Top-most enriched processes and pathways of the parental genes of severe COVID-19 specific recurrent chimeric transcripts detected by Metascape (*p*-value cutoff < 0.05).

Chimeric transcripts expressed in multiple severe COVID-19 patient blood samples could be associated with the development of the severity of COVID-19. We have identified 42 chimeric transcripts as recurrent, which are expressed in at least 3 severe COVID-19 patient samples (Table S2). The generation of chimeric transcripts could impact on the functionality of their parental genes [27,49]. For example, chimeric transcripts could translate into chimeric proteins that could replace the interactions of their parental proteins in the PPI network and alter the functionality of the cell [27,29,50]. Furthermore, chimeric transcripts could act as long non-coding RNA (lncRNA), which could regulate and alter the functionality of their parental genes [49,51,52]. We have found 75 parental genes generating recurrent chimeric transcripts in severe COVID-19 patients. So, it can be assumed that severe COVID-19 specific, recurrent chimeric transcripts could impact on the functionality of their parental genes, which may lead to the development of disease severity. We performed the GO functional and pathway enrichment analysis to understand the potentiality for alteration of chimeric transcripts mediated by cellular functionality. A Metascape analysis of the most enriched biological processes revealed that the parental genes of chimeric transcripts are involved in a broad range of important processes such as localization, cellular processes, immune system processes, metabolic processes, regulation of biological processes, etc. (Figure 2A). This observation indicates that chimeric transcripts generated from these genes could play a potential role in alternations of immune-related processes and alter the regulation of the diverse biological and metabolic processes that led to the development of disease severity. Further, the top-most enriched pathway and process analysis revealed that cellular response to stress, phagocytosis, and interferon alpha/beta signaling are the most important processes that could be altered by these chimeric transcripts (Figure 2B).

### 3.3. Genomic Neighborhood Analysis and Differential Gene Expression Analysis of the Parental Genes of Severe COVID-19 Specific Recurrent Chimeric Transcripts

**Figure 3:**
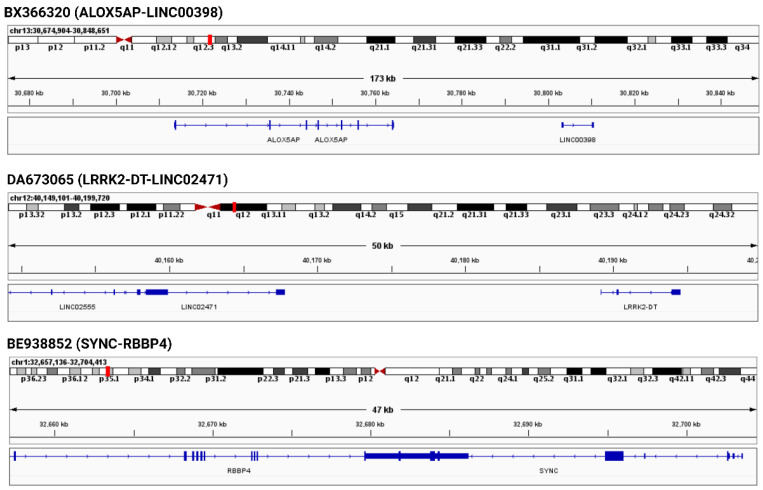
Genomic neighborhood analysis of the parental genes of severe COVID-19 specific recurrent chimeric transcripts. The two parental genes of chimeric transcripts adjacent to each other indicate the potentiality of cis-SAGe.

Several chimeric transcripts could be generated by the non-canonical splicing-based mechanisms, such as the trans-splicing and cis-splicing of adjacent genes (cis-SAGe) [25]. Trans-splicing is the mechanism by which two transcripts from two different genes could fuse and generate a novel chimeric transcript [53,54,55,56]. However, the mechanism of trans-splicing events is not well understood in humans, and no such computational method exists that can predict whether a chimeric transcript is generated by a trans-splicing mechanism. cis-SAGe is a frequent mechanism in human cancers and other complex diseases in which this mechanism generates a significant number of chimeric transcripts [17,18,57,58,59]. However, a recent study demonstrated that cis-SAGe is the common mechanism for generating chimeric transcripts in healthy human cells [17,22]. cis-SAGe is the intergenic splicing of directly adjacent genes with the same transcriptional orientation, which can generate chimeric transcripts [60]. We performed the genomic neighborhood analysis of the parental genes from severe COVID-19 specific recurrent chimeric transcripts, and we found seven confirmed cases where those chimeric transcripts were generated by cis-SAGe analysis (Table S2). Integrative Genomics Viewer (IGV) [61] was used to visualize the adjacent genes. Figure 3 and Figure S1 support the close proximity of the two genes from different recurrent severe COVID-19 specific chimeric transcripts and highlight the potentiality of cis-SAGe mechanism.

**Figure 4:**
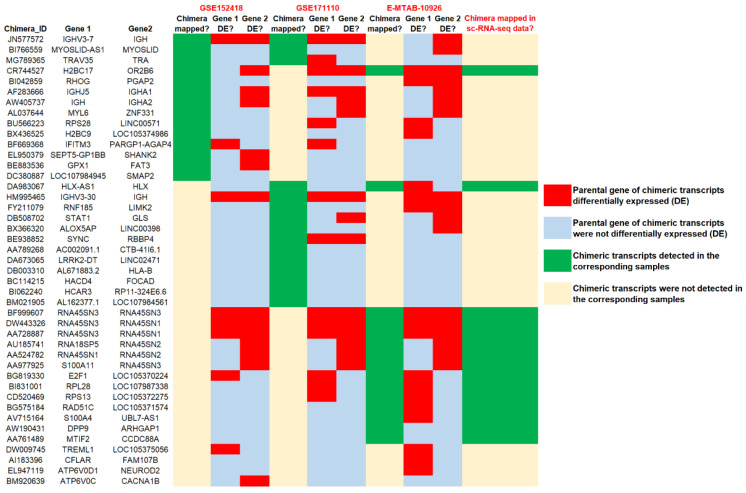
Differential gene expression analysis of the parental genes of severe COVID-19 specific recurrent chimeric transcripts.

If the emergence of chimeric transcripts can regulate the functionality of their parental genes, then we might expect the differential expression of at least one of their parental genes. Interestingly, we observed in several instances that at least one of the parental genes of recurrent severe COVID-19 specific chimeric transcripts from different datasets were differentially expressed (Figure 4). This finding could suggest that chimeric transcripts generated in severe COVID-19 could significantly regulate their parental genes and alter the pathways and biological processes where their parental genes are involved.

### 3.4. Identification of Common Chimeric Transcripts Expressed in Severe COVID-19 and Other Severe Respiratory Viral Infections

**Figure 5:**
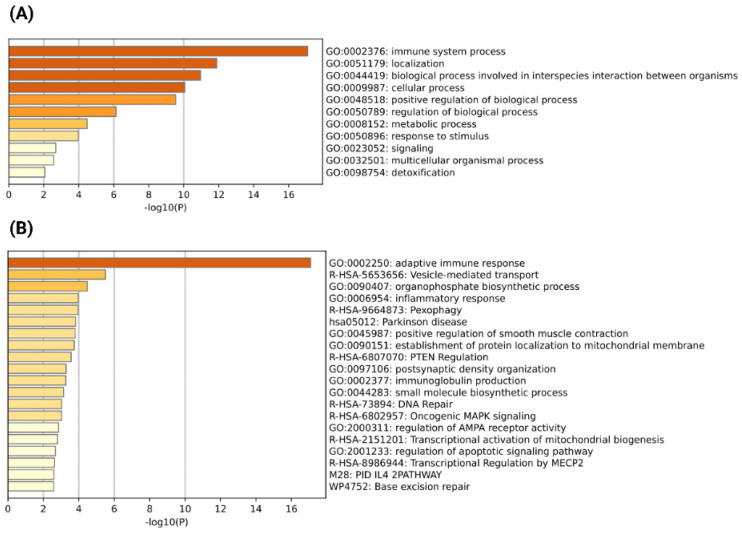
(**A**) GO biological process enrichment analysis of the parental genes of common chimeric transcripts associated with multiple severe respiratory viral infections and severe COVID-19; (**B**) Top-most enriched processes and pathways of the parental genes of common chimeric transcripts associated with multiple severe respiratory viral infections and severe COVID-19 detected by Metascape (*p*-value cutoff < 0.05).

Common chimeric transcripts from multiple severe respiratory viral infections and severe COVID-19 could be associated with the immune response common to the development of disease severity caused by most respiratory viral infections. With this aim, we have identified 189 common chimeric transcripts unique to both severe COVID-19 and multiple other severe respiratory viral infections (Table S3). Then, we analyzed the functional and pathway enrichment analysis of the parental genes of these common chimeric transcripts. In line with the GO biological process enrichment of the parental genes of recurrent severe COVID-19 specific chimeric transcripts, here we observed the parental genes of common chimeric transcripts of severe COVID-19 infections and other severe respiratory viral infections are involved in important processes related to immune system processes, localization, biological processes involved in interspecies interaction, cellular processes, metabolic processes, regulation of biological processes, etc. (Figure 5A). This observation suggests that the chimeric transcripts mostly generated in severe viral infections are crucial for immune dysfunctions and alternations of various important cellular, metabolic, and regulatory processes and pathways leading to the development of disease severity. From the top-most enriched biological process and pathway analysis, we found the adaptive immune response is the most important process (Figure 5B), which indicates that these chimeric transcripts generated from these genes could significantly associate with the adaptive immune response. Furthermore, the inflammatory response was found to be another enriched process which is an important factor in viral infection-mediated disease severity (Figure 5B). Next, we observed the significant enrichment of the vesicle-mediated transport pathway (Figure 5B), which suggests that the chimeric transcripts could be associated with the transport of viral particles into the membrane-bounded vesicles, which is an efficient mechanism for viruses to enter host cells and evade the host immune response [62,63,64]. This line of observations supports our hypothesis that the chimeric transcripts common to multiple severe respiratory viral infections, including COVID-19, could impact the various important biological processes and pathways that drive the stress responses leading to the development of disease severity.

## 4. Discussion

In this study, we analyzed several publicly available RNA-seq datasets containing 85 severe COVID-19 patients’ blood samples, 10 mild COVID-19 patients’ blood samples, 84 other respiratory viral infected patients’ blood samples, and 253 healthy human samples from 32 different tissues, including blood. We identified 424 severe COVID-19 specific chimeric transcripts; among these, 42 chimeric transcripts were recurrent which were mapped in at least 3 samples. The important enriched biological processes and pathways of the parental genes of these recurrent severe COVID-19 specific chimeric transcripts highlighted that they are involved in cellular stress response, phagocytosis, and interferon signaling. The genes associated with the cellular stress response pathway could be involved in the activation of innate and adaptive immune responses and play a pivotal role in host defenses and inflammation [65]. Next, we observed the second most enriched process/pathway is phagocytosis. Recent studies demonstrated that alternations of the phagocytosis response are strongly associated with COVID-19 severity [66,67,68]. However, the molecular mechanisms behind the phagocytosis alternations and COVID-19 severity development were not clearly understood. Our findings suggest that the recurrent severe COVID-19 specific chimeric transcripts could alter the functionality of their parental genes which are involved in the phagocytosis process. Further, we observed that parental genes of the recurrent severe COVID-19-specific chimeric transcripts were significantly involved in interferon signaling. Recent studies showed that the dysregulated interferon response is crucial for the development of severe COVID-19 [69,70]. Interferon signaling induces the IFN-stimulated gene (ISG) expression by phosphorylating STAT1, and STAT1 phosphorylation was found to increase in severe COVID-19 cases, indicating an imbalanced JAK/STAT signaling and lack of ISG-induced transcription [69]. In this study, we observed that the cis-SAGe mechanism STAT1 gene generated a recurrent severe COVID-19 specific chimeric transcript. Altogether, these findings could suggest the potential involvement of recurrent severe COVID-19 specific chimeric transcripts in the generation of a stress response associated with dysregulated interferon signaling during severe COVID-19 infections.

If the chimeric transcripts alter the functionality of their parental genes, then we should expect at least one of their parental genes could be differentially expressed. Interestingly, we observed in several instances that one of the parental genes of recurrent severe COVID-19 specific chimeric transcripts was differentially expressed. We observed consistent differential gene expression patterns for some chimeric transcript’s parental genes in all datasets; however, the chimeric transcripts mapped in some datasets, but not all (Figure 4). We chose strict criterion (Materials and Methods (2.2)) for chimeric transcripts detection to reduce the chances of obtaining false positives. So, as the quality of the RNA-seq data is different in each dataset, we could have missed some chimeric transcripts because fewer reads were mapped, or mapping read qualities were low. Similarly, the number of differentially expressed genes in each dataset depends on the quality of sequencing data and the *p*-value criteria selected to screen the differentially expressed genes. We chose the strict *p*-value, *p* <0.05, as the criterion for detection of differential gene expression, so there is a chance that we could have missed some differentially expressed genes in some datasets because of the quality of the sequencing data and read count bias. Interestingly, instead of the difference of sequence quality in different datasets, we mapped all the recurrent severe COVID-19 specific chimeric transcripts detected in the E-MTAB-10926 samples in the single-cell high-quality (scRNA)-seq dataset from the PRJNA633393 samples. Therefore, the production of the chimeric transcripts could be the important signature behind the generation of stress responses leading to COVID-19 severity development. Furthermore, we found 189 common chimeric transcripts unique to both severe COVID-19 and other severe respiratory viral infections whose parental genes were enriched in adaptive immune response and inflammatory response. This could signify these chimeric transcripts might be associated with stress responses associated with dysfunctional immune responses common to multiple respiratory viral severe infections.

**Figure 6:**
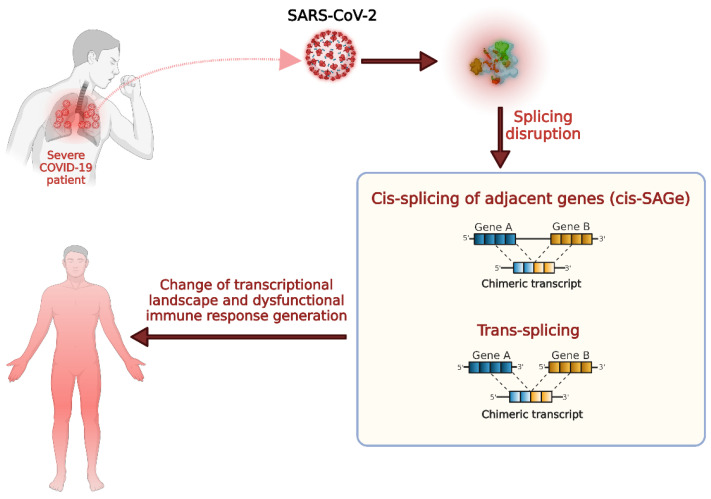
Schematic representation of the generation of chimeric transcripts in severe COVID-19 and their potential functional impact.

Our study first identified the chimeric transcripts unique to severe COVID-19 infections and demonstrated their potential functional importance (Figure 6). During a severe COVID-19 infection, SARS-CoV-2 targets the host cell, splicing to alter the transcriptional dynamics of the host cell so that they can efficiently translate the viral proteins and evade the host cell’s defense mechanisms. The aberrant splicing during severe COVID-19 infections could promote the generation of chimeric transcripts that can alter the function of their parental genes, which are mainly associated with immune-related processes that could lead to the development of immune dysfunction and disease severity development. Therefore, further exploring the functional role of individual recurrent severe COVID-19 specific chimeric transcripts could aid in understanding the individual’s immune responses to COVID-19 infection, which might help to design personalized treatment strategies.

## 5. Conclusions

The findings of this present study suggest the potential role of recurrent severe COVID-19 specific chimeric transcripts in alternations of immune-related processes, activating the processes that favor the viral transcription, gene expression, and alternations of various biological and metabolic processes that could lead to the development of severe disease. Furthermore, we identified common chimeric transcripts found in severe COVID-19 and other severe respiratory viral infections. The parental genes of these common chimeric transcripts are functionally enriched for adaptive immune response and inflammatory response. These observations suggest these chimeric transcripts could be associated with different stresses associated with the dysfunctional immune response generated during multiple severe respiratory viral infections, including COVID-19. The limitation of our study was the lack of experimental validation, as we relied on publicly available RNA-seq data from patients. Further, the study’s use of publicly available datasets had varying experimental designs, and the patient samples came from diverse populations which were collected at different time points during the development of COVID-19 severity. Despite the limitations inherent to computational analysis, this study successfully uncovered the hidden layers of the blood transcriptome from the various respiratory viral infected patients and identified several potential functionally relevant chimeric transcripts unique to severe COVID-19 and common to multiple severe respiratory virus infections. Further experimental studies are necessary to elucidate the specific functions of these chimeric transcripts in COVID-19 pathogenesis.

## Data Availability

All the data supported our findings have been provided in the supplementary materials.

## Supplementary Materials

The following are available online at https://www.mdpi.com/article/10.3390/v15020433/s1, Table S1: Chimeric transcripts detected in all the samples; Table S2: List of recurrent severe COVID-19 specific chimeric transcripts which were detected in at least three severe COVID-19 patients’ blood samples; Table S3: List of common chimeric transcripts unique to both severe COVID-19 and other severe respiratory viral infections; Figure S1: Genomic neighborhood analysis of the parental genes of severe COVID-19 specific recurrent chimeric transcripts.

## References

[1] Wu F., Zhao S., Yu B., Chen Y.M., Wang W., Song Z.G., Hu Y., Tao Z.W., Tian J.H., Pei Y.Y. (2020). A new coronavirus Correctassociated with human respiratory disease in China. Nature.

[2] Lai C.C., Shih T.P., Ko W.C., Tang H.J., Hsueh P.R. (2020). Severe acute respiratory syndrome coronavirus 2 (SARS-CoV-2) and coronavirus disease-2019 (COVID-19): The epidemic and the challenges. Int. J. Antimicrob. Agents.

[3] Channappanavar R., Perlman S. (2017). Pathogenic human coronavirus infections: Causes and consequences of cytokine storm and immunopathology. Semin. Immunopathol..

[4] Fan E., Beitler J.R., Brochard L., Calfee C.S., Ferguson N.D., Slutsky A.S., Brodie D. (2020). COVID-19-associated acute respiratory distress syndrome: Is a different approach to management warranted?. Lancet Respir. Med..

[5] Berlin D.A., Gulick R.M., Martinez F.J. (2020). Severe COVID-19. N. Engl. J. Med..

[6] Ortiz-Prado E., Simbaña-Rivera K., Gómez- Barreno L., Rubio-Neira M., Guaman L.P., Kyriakidis N.C., Muslin C., Jaramillo A.M.G., Barba-Ostria C., Cevallos-Robalino D. (2020). Clinical, molecular, and epidemiological characterization of the SARS-CoV-2 virus and the Coronavirus Disease 2019 (COVID-19), a comprehensive literature review. Diagn. Microbiol. Infect. Dis..

[7] Rockx B., Kuiken T., Herfst S., Bestebroer T., Lamers M.M., Munnink B.B.O., De Meulder D., Van Amerongen G., Van Den Brand J., Okba N.M.A. (2020). Comparative pathogenesis of COVID-19, MERS, and SARS in a nonhuman primate model. Science.

[8] Mukherjee S., Banerjee B., Karasik D., Frenkel-Morgenstern M. (2021). mRNA-lncRNA Co-Expression Network Analysis Reveals the Role of lncRNAs in Immune Dysfunction during Severe SARS-CoV-2 Infection. Viruses.

[9] Vaninov N. (2020). In the eye of the COVID-19 cytokine storm. Nat. Rev. Immunol..

[10] Coperchini F., Chiovato L., Croce L., Magri F., Rotondi M. (2020). The cytokine storm in COVID-19: An overview of the involvement of the chemokine/chemokine-receptor system. Cytokine Growth Factor Rev..

[11] Hojyo S., Uchida M., Tanaka K., Hasebe R., Tanaka Y., Murakami M., Hirano T. (2020). How COVID-19 induces cytokine storm with high mortality. Inflamm. Regen..

[12] Leisman D.E., Ronner L., Pinotti R., Taylor M.D., Sinha P., Calfee C.S., Hirayama A.V., Mastroiani F., Turtle C.J., Harhay M.O. (2020). Cytokine elevation in severe and critical COVID-19: A rapid systematic review, meta-analysis, and comparison with other inflammatory syndromes. Lancet Respir. Med..

[13] Banerjee A.K., Blanco M.R., Bruce E.A., Honson D.D., Chen L.M., Chow A., Bhat P., Ollikainen N., Quinodoz S.A., Loney C. (2020). SARS-CoV-2 Disrupts Splicing, Translation, and Protein Trafficking to Suppress Host Defenses. Cell.

[14] Wang C., Chen L., Chen Y., Jia W., Cai X., Liu Y., Ji F., Xiong P., Liang A., Liu R. (2022). Abnormal global alternative RNA splicing in COVID-19 patients. PLoS Genet..

[15] Mukherjee S.B., Mukherjee S., Detroja R., Frenkel-Morgenstern M. (2023). The landscape of differential splicing and transcript alternations in severe COVID-19 infection. FEBS J..

[16] Sveen A., Kilpinen S., Ruusulehto A., Lothe R.A., Skotheim R.I. (2016). Aberrant RNA splicing in cancer; Expression changes and driver mutations of splicing factor genes. Oncogene.

[17] Zhuo J.-S., Jing X.-Y., Du X., Yang X.-Q. (2018). Generation of Chimeric RNAs by cis-splicing of adjacent genes (cis-SAGe) in mammals. Yi Chuan Hered..

[18] Jia Y., Xie Z., Li H. (2016). Intergenically Spliced Chimeric RNAs in Cancer. Trends Cancer.

[19] Sibley C.R., Blazquez L., Ule J. (2016). Lessons from non-canonical splicing. Nat. Rev. Genet..

[20] Shi X., Singh S., Lin E., Li H. (2021). Chimeric RNAs in cancer. Advances in Clinical Chemistry.

[21] Pitolli C., Marini A., Sette C., Pagliarini V. (2022). Non-Canonical Splicing and Its Implications in Brain Physiology and Cancer. Int. J. Mol. Sci..

[22] Chwalenia K., Facemire L., Li H. (2017). Chimeric RNAs in cancer and normal physiology. Wiley Interdiscip. Rev. RNA.

[23] Mukherjee S., Heng H.H., Frenkel-Morgenstern M. (2021). Emerging Role of Chimeric RNAs in Cell Plasticity and Adaptive Evolution of Cancer Cells. Cancers.

[24] Detroja R., Mukherjee S., Frenkel-Morgenstern M. (2022). The Landscape of Novel Expressed Chimeric RNAs in Rheumatoid Arthritis. Cells.

[25] Mukherjee S., Frenkel-Morgenstern M. (2022). Evolutionary impact of chimeric RNAs on generating phenotypic plasticity in human cells. Trends Genet..

[26] Mukherjee S., Mukherjee S.B., Frenkel-Morgenstern M. (2023). Functional and regulatory impact of chimeric RNAs in human normal and cancer cells. Wiley Interdiscip. Rev. RNA.

[27] Mukherjee S.B., Mukherjee S., Frenkel-Morgenstern M. (2022). Fusion proteins mediate alternation of protein interaction networks in cancers. Advances in Protein Chemistry and Structural Biology.

[28] Frenkel-Morgenstern M., Lacroix V., Ezkurdia I., Levin Y., Gabashvili A., Prilusky J., Del Pozo A., Tress M., Johnson R., Guigo R. (2012). Chimeras taking shape: Potential functions of proteins encoded by chimeric RNA transcripts. Genome Res..

[29] Latysheva N.S., Babu M.M. (2019). Molecular Signatures of Fusion Proteins in Cancer. ACS Pharmacol. Transl. Sci..

[30] Lévy Y., Wiedemann A., Hejblum B.P., Durand M., Lefebvre C., Surénaud M., Lacabaratz C., Perreau M., Foucat E., Déchenaud M. (2021). CD177, a specific marker of neutrophil activation, is associated with coronavirus disease 2019 severity and death. iScience.

[31] Arunachalam P.S., Wimmers F., Mok C.K.P., Perera R.A.P.M., Scott M., Hagan T., Sigal N., Feng Y., Bristow L., Tsang O.T.Y. (2020). Systems biological assessment of immunity to mild versus severe COVID-19 infection in humans. Science.

[32] Wilk A.J., Rustagi A., Zhao N.Q., Roque J., Martínez-Colón G.J., McKechnie J.L., Ivison G.T., Ranganath T., Vergara R., Hollis T. (2020). A single-cell atlas of the peripheral immune response in patients with severe COVID-19. Nat. Med..

[33] Jackson H., Rivero Calle I., Broderick C., Habgood-Coote D., D’Souza G., Nichols S., Vito O., Gómez-Rial J., Rivero-Velasco C., Rodríguez-Núñez N. (2022). Characterisation of the blood RNA host response underpinning severity in COVID-19 patients. Sci. Rep..

[34] Tsalik E.L., Fiorino C., Aqeel A., Liu Y., Henao R., Ko E.R., Burke T.W., Reller M.E., Bodinayake C.K., Nagahawatte A. (2021). The Host Response to Viral Infections Reveals Common and Virus-Specific Signatures in the Peripheral Blood. Front. Immunol..

[35] Leinonen R., Sugawara H., Shumway M. (2011). The sequence read archive. Nucleic Acids Res..

[36] Detroja R., Gorohovski A., Giwa O., Baum G., Frenkel-Morgenstern M. (2021). ChiTaH: A fast and accurate tool for identifying known human chimeric sequences from high-throughput sequencing data. NAR Genom. Bioinform..

[37] Benelli M., Pescucci C., Marseglia G., Severgnini M., Torricelli F., Magi A. (2012). Discovering chimeric transcripts in paired-end RNA-seq data by using EricScript. Bioinformatics.

[38] Haas B., Dobin A., Stransky N., Li B., Yang X., Tickle T., Bankapur A., Ganote C., Doak T., Pochet N. (2017). STAR-Fusion: Fast and Accurate Fusion Transcript Detection from RNA-Seq. bioRxiv.

[39] Davidson N.M., Majewski I.J., Oshlack A. (2015). JAFFA: High sensitivity transcriptome-focused fusion gene detection. Genome Med..

[40] Nicorici D., Satalan M., Edgren H., Kangaspeska S., Murumagi A., Kallioniemi O., Virtanen S., Kilkku O. (2014). FusionCatcher—A tool for finding somatic fusion genes in paired-end RNA-sequencing data. bioRxiv.

[41] Balamurali D., Gorohovski A., Detroja R., Palande V., Raviv-Shay D., Frenkel-Morgenstern M. (2019). ChiTaRS 5.0: The comprehensive database of chimeric transcripts matched with druggable fusions and 3D chromatin maps. Nucleic Acids Res..

[42] Dobin A., Davis C.A., Schlesinger F., Drenkow J., Zaleski C., Jha S., Batut P., Chaisson M., Gingeras T.R. (2013). STAR: Ultrafast universal RNA-seq aligner. Bioinformatics.

[43] Liao Y., Smyth G.K., Shi W. (2014). featureCounts: An efficient general purpose program for assigning sequence reads to genomic features. Bioinformatics.

[44] Love M.I., Anders S., Huber W. (2014). Differential analysis of count data—The DESeq2 package. Genome Biol..

[45] Haynes W. (2013). Benjamini–Hochberg Method. Encyclopedia of Systems Biology.

[46] Zhou Y., Zhou B., Pache L., Chang M., Khodabakhshi A.H., Tanaseichuk O., Benner C., Chanda S.K. (2019). Metascape provides a biologist-oriented resource for the analysis of systems-level datasets. Nat. Commun..

[47] Kanehisa M., Goto S. (2000). KEGG: Kyoto Encyclopedia of Genes and Genomes. Nucleic Acids Res..

[48] Fabregat A., Jupe S., Matthews L., Sidiropoulos K., Gillespie M., Garapati P., Haw R., Jassal B., Korninger F., May B. (2018). The Reactome Pathway Knowledgebase. Nucleic Acids Res..

[49] Mukherjee S., Detroja R., Balamurali D., Matveishina E., Medvedeva Y.A., Valencia A., Gorohovski A., Frenkel-Morgenstern M. (2021). Computational analysis of sense-antisense chimeric transcripts reveals their potential regulatory features and the landscape of expression in human cells. NAR Genom. Bioinform..

[50] Frenkel-Morgenstern M., Gorohovski A., Tagore S., Sekar V., Vazquez M., Valencia A. (2017). ChiPPI: A novel method for mapping chimeric protein-protein interactions uncovers selection principles of protein fusion events in cancer. Nucleic Acids Res..

[51] Qin F., Zhang Y., Liu J., Li H. (2017). SLC45A3-ELK4 functions as a long non-coding chimeric RNA. Cancer Lett..

[52] Guo M., Xiao Z.D., Dai Z., Zhu L., Lei H., Diao L.T., Xiong Y. (2020). The landscape of long noncoding RNA-involved and tumor-specific fusions across various cancers. Nucleic Acids Res..

[53] Sun Y., Li H. (2022). Chimeric RNAs Discovered by RNA Sequencing and Their Roles in Cancer and Rare Genetic Diseases. Genes.

[54] Wang Y., Zou Q., Li F., Zhao W., Xu H., Zhang W., Deng H., Yang X. (2021). Identification of the cross-strand chimeric RNAs generated by fusions of bi-directional transcripts. Nat. Commun..

[55] Li H., Wang J., Mor G., Sklar J. (2008). A neoplastic gene fusion mimics trans-splicing of RNAs in normal human cells. Science.

[56] Jividen K., Li H. (2014). Chimeric RNAs generated by intergenic splicing in normal and cancer cells. Genes Chromosom. Cancer.

[57] Qin F., Song Z., Babiceanu M., Song Y., Facemire L., Singh R., Adli M., Li H. (2015). Discovery of CTCF-Sensitive Cis-Spliced Fusion RNAs between Adjacent Genes in Human Prostate Cells. PLoS Genet..

[58] Qin F., Song Y., Zhang Y., Facemire L., Frierson H., Li H. (2016). Role of CTCF in regulating SLC45A3-ELK4 chimeric RNA. PLoS One.

[59] Qin F., Song Z., Chang M., Song Y., Frierson H., Li H. (2016). Recurrent cis-SAGe chimeric RNA, D2HGDH-GAL3ST2, in prostate cancer. Cancer Lett..

[60] Zhang Y., Gong M., Yuan H., Park H.G., Frierson H.F., Li H. (2012). Chimeric transcript generated by cis- splicing of adjacent genes regulates prostate cancer cell proliferation. Cancer Discov..

[61] Thorvaldsdóttir H., Robinson J.T., Mesirov J.P. (2013). Integrative Genomics Viewer (IGV): High-performance genomics data visualization and exploration. Brief. Bioinform..

[62] Bello-Morales R., Ripa I., López-Guerrero J.A. (2020). Extracellular vesicles in viral spread and antiviral response. Viruses.

[63] Meckes D.G., Raab-Traub N. (2011). Microvesicles and Viral Infection. J. Virol..

[64] van Dongen H.M., Masoumi N., Witwer K.W., Pegtel D.M. (2016). Extracellular Vesicles Exploit Viral Entry Routes for Cargo Delivery. Microbiol. Mol. Biol. Rev..

[65] Muralidharan S., Mandrekar P. (2013). Cellular stress response and innate immune signaling: Integrating pathways in host defense and inflammation. J. Leukoc. Biol..

[66] Kvedaraite E., Hertwig L., Sinha I., Ponzetta A., Myrberg I.H., Lourda M., Dzidic M., Akber M., Klingström J., Folkesson E. (2021). Major alterations in the mononuclear phagocyte landscape associated with COVID-19 severity. Proc. Natl. Acad. Sci. USA.

[67] Tay M.Z., Poh C.M., Rénia L., MacAry P.A., Ng L.F.P. (2020). The trinity of COVID-19: Immunity, inflammation and intervention. Nat. Rev. Immunol..

[68] Koenis D.S., Beegun I., Jouvene C.C., Aguirre G.A., Souza P.R., Gonzalez-Nunez M., Ly L., Pistorius K., Kocher H.M., Ricketts W. (2021). Disrupted Resolution Mechanisms Favor Altered Phagocyte Responses in COVID-19. Circ. Res..

[69] Eskandarian Boroujeni M., Sekrecka A., Antonczyk A., Hassani S., Sekrecki M., Nowicka H., Lopacinska N., Olya A., Kluzek K., Wesoly J. (2022). Dysregulated Interferon Response and Immune Hyperactivation in Severe COVID-19: Targeting STATs as a Novel Therapeutic Strategy. Front. Immunol..

[70] Soltani-Zangbar M.S., Parhizkar F., Ghaedi E., Tarbiat A., Motavalli R., Alizadegan A., Aghebati-Maleki L., Rostamzadeh D., Yousefzadeh Y., Jadideslam G. (2022). A comprehensive evaluation of the immune system response and type-I Interferon signaling pathway in hospitalized COVID-19 patients. Cell Commun. Signal..

