# Supplementary figures and images for: The Landscape of Expressed Chimeric Transcripts in the Blood of Severe COVID-19 Infected Patients
Source: Viruses. 2023 Feb 4;15(2):433. doi: 10.3390/v15020433 (PMC9958880; doi:10.3390/v15020433)

BI042859 (RHOG-PGAP2)

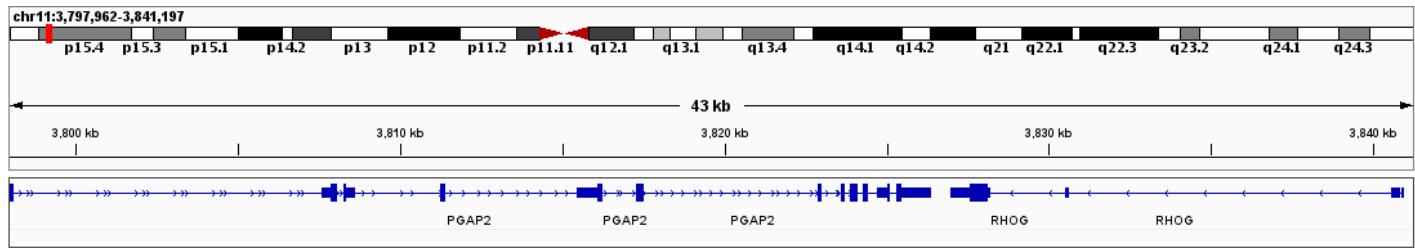

DA983067 (HLX-AS1-HLX)

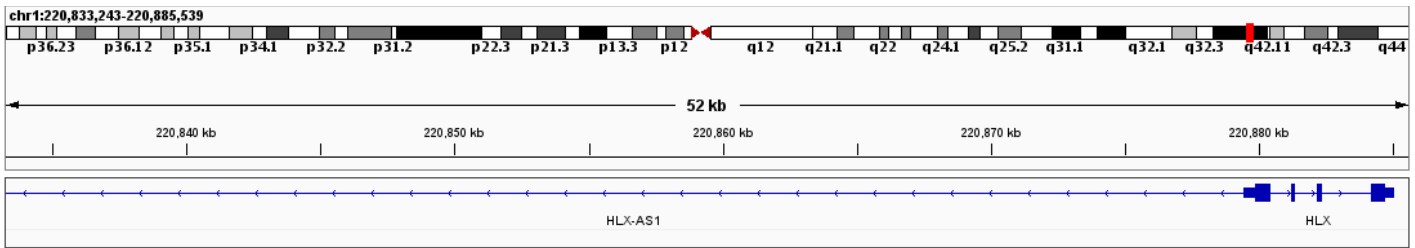

DB508702 (STAT1-GLS)

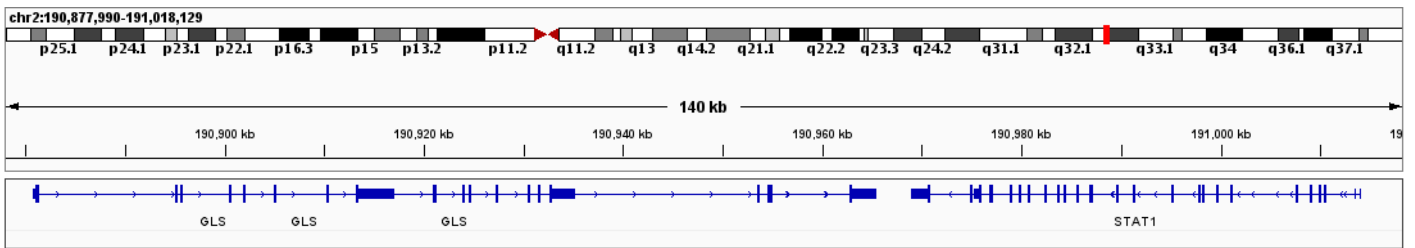

FY211079:RNF185-LIMK2

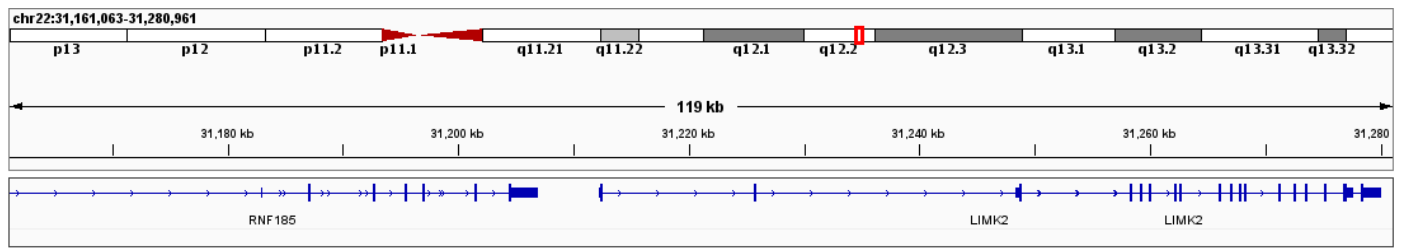

Supplement: Supplementary file 1 [file viruses-15-00433-s001.zip › Figure S1.pdf]
